# The hybrid approach for the surgical treatment of lone atrial fibrillation: One-year results employing a monopolar radiofrequency source

**DOI:** 10.1186/1749-8090-7-71

**Published:** 2012-07-19

**Authors:** Mark La Meir, Sandro Gelsomino, Roberto Lorusso, Fabiana Lucà, Laurant Pison, Orlando Parise, Francis Wellens, Gian Franco Gensini, Jos Maessen

**Affiliations:** 1 University Hospital Maastricht, Maastricht, The Netherlands; 2 Careggi Hospital, Florence, Italy; 3 Civic Hospital Brescia, Brescia, Italy; 4 University Hospital Brussels, Brussels, Belgium

**Keywords:** Atrial fibrillation, Lone atrial fibrillation, Ablation, Minimally invasive

## Abstract

**Background:**

The hybrid technique combines a mono or bilateral epicardial approach with a percutaneous endocardial ablation in a single-step procedure. We present our early results with this technique employing a monopolar radiofrequency source through a right thoracoscopy in patients with lone atrial fibrillation (LAF).

**Methods:**

Between June 2009 and December 2010 nineteen consecutive patients (mean 60.8 ± 8.6 years, 84.2% male) underwent right unilateral minimally invasive hybrid procedure for LAF at our Institution. Ten patients (52.6.6%) had long-standing persistent AF while four (21.1%) had persistent and five (26.3%) paroxysmal AF. All patients were followed-up according the Heart Rhythm Society/European Heart Rhythm Association/European Cardiac Arrhythmia Society (HRS/EHRA/ECA) and Society of Thoracic Surgeon (STS) guidelines.

**Results:**

There were neither early nor late deaths. It was possible to complete all the procedures as planned without any conversion to cardiopulmonary bypass. No patient died during the follow up. At one year, 7/19 (36.8%) patients were in sinus rhythm with no episode of AF and off antiarrhythmic drugs (AAD). Time-related prevalence of postoperative AF peaked at 44.4% (41.3–47.4) at two weeks, was 30.4% (27.3–34.9) at three months, fell to 14.2% (11.6–18.1) by 6 months and was 13.3% (11.0–17.4) at 12 months Among patients with long-standing persistent (LSP) AF, 20% (2/10) were in Sinus rythm and off AAD. One-year success rates were 50% (2/4) in persistent and 60% (3/5) in paroxysmal AF. At 12 months estimated prevalence of antyarrhythmic drugs and Warfarin use were 26% (22.4–33.1) and 48% (37.2–53.2), respectively.

**Conclusions:**

One year results combining the percutaneous endocardial with the right thoracoscopic epicardial technique were, in our experience, not satisfactory, particularly in patients with LSP and persistent AF. Our findings need to be confirmed by larger studies.

## Background

Despite being more effective than percutaneous catheter ablation (PCA) [1]-3], the MAZE operation failed to achieve widespread application as stand-alone procedure because of its complexity and invasiveness. Indeed, the Society for Thoracic Surgeons (STS) database [4] reported, in 2007, only 700 MAZE operations performed in patients with lone atrial fibrillation (LAF). In addiition, along with its suboptimal success rates, PCA showed a non-negligible incidence of major complications [3]. New technologies have allowed the creation of transmural lesions on a beating heart through alternative, less invasive incisions [5]. Nonetheless, results reported in the literature with these approaches are highly variable [6-8].

More recently, a sequential one-step surgical/catheter-based approach has been introduced showing satisfactory results in patients with persistent LAF [9]. This so-called hybrid procedure combines the advantages of PCA and video-assisted thoracoscopic epicardial procedures and it is expected to overcome the shortcomings of these techniques [10].

As originally described, the hybrid technique employs a bilateral thoracoscopic epicardial approach. We have recently introduced a less-invasive hybrid single-sided approach through a right thoracoscopy and, as far as we know, there are no reports in the literature about the effectiveness of this procedure.

Therefore, we present one-year results with minimally invasive hybrid-right thoracoscopic approach employing a monopolar radiofrequency (RF) source for the treatment of LAF.

## Methods

### Patients

Ethical Committee approval was waived according to the National law regulating observational retrospective studies (Dutch WMO law). However, all patients gave their informed consent to access their data for scientific purposes.

Between January 2008 and June 2010 nineteen consecutive patients underwent minimally invasive right-thoracoscopic hybrid ablation of LAF employing a monopolar RF source. LAF was defined as suggested by American College of Cardiology/American Heart Association/European Society of Cardiology (ACC/AHA/ESC) Guidelines [11] and updated ESC Guidelines were followed to distinguish the type of AF and to score the AF-related symptoms (European Heart Rhythm Association [EHRA] score) [12]. Indication for minimally invasive surgery was based on the Heart Rhythm Society/European Heart Rhythm Association/European Cardiac Arrhythmia Society (HRS/EHRA/ECA) Guidelines [13]. A trans-thoracic echocardiography (TTE) and a computed tomography (CT) scan were carried out preoperatively (pulmonary vein anatomy, coronary arteries) and potential candidates for the hybrid procedure underwent a lung function test (spirometry). Exclusion criteria were: 1) Presence of left atrial or left appendage thrombus at transesophageal echocardiography (TEE); 2) ″Giant″ left atrium (diameter >6.5 cm); 3) Associated coronary artery disease; 4) Previous pulmonary or cardiac surgery. Patient characteristics are shown in Table 1.

**Table 1:** Baseline Patient Characteristics (n = 19)

Age	61.2 ± 8.6
M/F	16/3 (84.2/15.8)
BMI	27.6 ± 4.6
Hypertension	7 (36.8)
TIA/CVA	1 (5.2)
Preoperative catheter ablation	
AF	4 (21.1)
Flutter	5 (26.3)
Type of preoperative AF	
Paroxysmal	5 (26.3)
Persistent	4 (21.1)
Long-standing persistent	10 (52.6)
Prevalence of AF (68% CI)	
Paroxysmal	20.0 (16.8–23.3)
Persistent	34.7 (31.2–38.9)
Long-standing persistent	63.6 (59.9–67.1)
EHRA Score	4 [3–4]
Duration of preoperative AF (yrs.)	5 [3–8.5]
Antyarrhythmic Drugs	
Amiodaron	2 (10.5)
Dysopiramide	1 (5.3)
Flecainide	6 (31.5)
Propaphenon	1 (5.3)
Sotalol	5 (26.3)
Electrical cardioversion	15 (78.9)
Previous Catheter Ablation	
For AF	6 (31.5)
For Atrial Flutter	5 (26.3)
Preoperative Pacemaker	2 (10.5)
Anticoagulant status	
Sodium Warfarin	16 (84.2)
Aspirin	5 (26.3)
LAVI (mL/m^2^)	47 ± 11
LA_MAX_ (mL/m^2^)	49 ± 20
LA_MIN_ (mL/m^2^)	30 ± 15
LA_EF_ (%)	38 ± 12
LA A-P (cm)	5.0 ± 0.5
LA S-I (cm)	6.4 ± 0.5

Normal data were presented as mean ±1 Standard deviation (SD), non parametric data as Median and [Interquartile Range] and discrete data as percentage (%).

**Abbreviations:** M/F: Male/Female; BMI: Body mass index; AF: Atrial fibrillation; CI: Confidence Interval; EHRA: European Hear Rhythm Association; LAVI: (Biplane) Left Atrial Volume Index; LA_MAX_: Maximum Left Atrial Volume; LA_MIN_: Minimum Left Atrial Volume; LA_EF_: Left Atrial emptying Fraction; LA A-P: Left Atrial Antero-Posterior Diameter; LA S-I : Left Atrial Superior-Inferior diameter.

### Follow-up and assessment of AF

All patients were followed-up according to Heart Rhythm Society/European Heart Rhythm Association/European Cardiac Arrhythmia Society (HRS/EHRA/ECA) [13]. Main outcomes were also reported following the Society of Thoracic Surgeon (STS) guidelines [14]. After hospital discharge patients underwent 7-day Holter Monitoring (HM) which was repeated at 3 months, 6 months and 1 year. All patients reached 1-year follow up. Monitoring was carried out with an external loop recorder (Del Mar Reynolds, Spacelabs Healthcare, Issaquah, WA,USA) and analyzed with Lifescreen Software (Del Mar Reynolds, Spacelabs Healthcare, Issaquah, WA, USA). For analysis, three rhythms were considered postoperative AF: AF, atrial flutter or atrial tachycardia lasting more than 30 sec. In addition, all electrocardiograms (ECG) performed at the discretion of referring physicians/cardiologists during the first three months after surgery and between Holter examinations were included when patients had at least two records available for analysis. Each ECG and Holter was treated as discrete data (presence/absence of atrial fibrillation) to calculate AF estimated prevalence [14]. A total of 222 postoperative Holter/ECG data were retrieved.

### Echocardiography

Echocardiography was performed preoperatively and at 3 month- and 12 month- follow-up appointments using a commercially available echocardiographic system (Philips iE33; Philips Medical Systems, Eindhoven, The Netherlands). All the parameters were analyzed “off-line” by an experienced echocardiographer (F.L.) using the Xcelera software (Philips Medical Systems Eindhoven, The Netherlands).

In the parasternal long-axis views LA maximum antero-posterior (A-P) diameter was measured. LA superior-inferior (S-I) diameter was measured from the mitral annular plane to the posterior wall of the LA in the apical 4-chamber view. In the apical 4-chamber view, LA maximum volume (LA max), at the end of LV systole, just before the opening of the mitral valve and LA minimum volume (LA min) at the end of LV diastole, just after the closure of the mitral valve, were measured [15]. LA emptying fraction (LA_EF_) was calculated as follows: (LA max-LA min/LA max) × 100. LA maximum volume was also measured by biplane area-length method [16] and indexed to body surface area (LAVI). LARR was defined as a reduction in LAVI ≥15% at late follow up [17].

### Anticoagulation and antiarrhythmic therapy

Antiarrhythmic drugs (AAD) were given postoperatively to all patients but, although we recommend discontinuing antyarrhythmics 3 months after ablation if the patients appears to be AF free, continued use is at the discretion of referring cardiologists.

Electrical cardioversion was not attempted for patients who remained in AF after the surgical procedure and was reserved for patients who were still in AF after 6 months.

Warfarin was administered on postoperative day II with INR target of 2.5 and stopped after 3 months if the Holter recording showed a sinus rhythm (SR) or patient had a low thromboembolic risk and a CHADS_2_ [cardiac failure, hypertension, age, diabetes, stroke (doubled)] score <2.

### Surgical technique

The interventions were performed under general anesthesia with a double-lumen endotracheal tube for selective lung ventilation. The chest was entered in the second, fourth and sixth interspaces using respectively a 5-12-12 mm port. The technique was as previously reported [18].

High frequency stimulation (HFS) of the right vagal nerve at the level of the mediastinum and at the four major ganglionated plexi (GPS, [right superior GP, right inferior GP, left superior GP and left inferior GP)] was carried out and the vagal response on the atrio-ventricular node was recorded (10 V, 1.5-ms pulse width impulse at 1000 pulses per minute from a temporary external pacemaker [Oscor, Oscor INC., Palm Harbor, FL]) The isolation of pulmonary veins was performed with a Cobra® Adhere XL (Estech, Danville, Ca) temperature-controlled, monopolar radiofrequency system. The ablation catheter was pushed under the superior caval vein (SCV) into the transverse sinus and placed behind the left atrial appendage (LAA) encircling the four PVs to create the box-lesion.

Before starting the ablation, the fat pad in the atrial groove was bluntly dissected to provide a better placement of the probe and enhance a higher penetration energy to destroy the GPs located inside this fat pad.

After creation of the box lesion the vagal response at level of GPS was tested again. The endpoint for ganglionated plexi (GP) ablation was the elimination of a vagal response to stimulation. In all patients this vagal response could no longer be induced with HFS, except for the right inferior GP which, being located outside the box lesion was not to ablated in any patient.

A left femoral vein puncture was made and a HIS bundle catheter (St Jude Medical, Inc, Minnetonka, MN, USA) and a coronary sinus (CS) catheter (Medtronic, Inc, Minneapolis, MN, USA) were placed under fluoroscopic guidance. Through the right femoral vein, a single trans-septal puncture was made using TEE and fluoroscopy and a long sheath 8 F (SL0, St. Jude Medical Daig Division, Inc., Minnetonka, MN, USA) was advanced into the LA. The patient was heparinzed to keep the activated clotting time >300 seconds. During rapid ventricular pacing from the HIS catheter, contrast was injected through the long sheath in order to visualize PVs and the LA. PVs were mapped with a suitably sized circular mapping catheter (Lasso, Biosense Webster, Inc, Diamond. Bar, CA, USA) placed at the ostium of the PVs.

In all patients epicardial pacing and endocardial recordings were utilized to demonstrate entrance and exit block; entrance block was defined as failure to capture the PVs during pacing from the left atrium at 18 V and 1.5-ms pulse width and the absence of pulmonary vein potentials (PVPs) seen on the Lasso catheter. Exit block was defined by failure to capture the left atrium during the pacing from the PVs and by failure to capture when pacing from each dipole of the Lasso catheter with an output of 10 mA and 2.0 ms pulse width.

We identified the conduction gaps from the endocardium which were closed endocardially with a 3.5 mm tip catheter (ThermoCool, Biosense Webster, Inc., Diamond. Bar, CA, USA) through the sheath in the LA. The precise location of the linear lesions was visualized with the Cobra® Adhere XL (Estech, Danville, Ca) in situ and using fluoroscopy. Gaps in linear lesions were defined as low amplitude and fragmented or narrowly split double atrial potentials.

In case of AF persistence, a left isthmus line was made endocardially from the mitral annulus towards the CS employing the ThermoCool catheter (ThermoCool, Biosense Webster, Inc., Diamond. Bar, CA, USA) The endpoint of ablation of the mitral isthmus was the bidirectional block which was achieved when the following criteria were met: 1) proximal-to-distal activation sequence along the CS catheter after pacing lateral to the line in the LA appendage; 2) Late activation on the opposite side after pacing on the septal side of the line through the CS; 3) widely separated local double potentials along the whole length of the ablation line. Finally, if the patient had a history of typical right atrial flutter or this arrhythmia became apparent during the procedure, we performed a cavo-tricuspid isthmus line (CTI) endocardially. The endpoint was bidirectional block and the presence of widely separated double potentials along the whole length of the ablation line.

### Statistical analysis

Normal values were expressed as mean ± one standard deviation (SD), non-normal values as median and interquartile range (IQR) and categorical variables as percentages. Student t, Wilcoxon and McNemar’s tests were employed where appropriate.

We analyzed all the intermittent data available in terms of time-related prevalence of AF [14] with a multiphase hazard decomposition method [19]. Prevalence of AF was presented as percentage with asymmetric 68% confidence obtained with the bootstrap percentile method [20].

Analyses of prevalence of AF do not account for antiarrhythmic medications. Prevalence of antiarrhythmic medication use was estimated by mixed modeling based on medication use at each follow-up assessment. With the same method we estimated the prevalence of Warfarin use at the time of each follow-up. Finally, the use of electrical cardioversion was analyzed as a repeated event and is presented as cumulative incidence [21].

Statistical analysis was performed using SPSS release 12.0 (SPSS, Chicago, IL, USA) and Curve Expert Professional release1.0.1 (D.G. Hyams, Chattanooga, TN, USA). *P* values less than 0.05 were considered significant.

## Results

None of the patients showed entrance and/or exit block after the epicardial ablation. Seventeen patients had at least one PV not isolated, which needed an endocardial touch-up (Table 2). After completing endocardial PV isolation the entire population had a conduction delay > 200 ms. in the posterior LA but no patient had complete block.

**Table 2:** Lesions set

**Left atrium**	
Right PVs Isolation	19 (100)
Left PVs Isolation	19 (100)
Inferior line	19 (100)
Roof line	19 (100)
Isthmus lesion	3 (15.7)
Endocardial gaps closure	17 (88.4)
**Right atrium**	
Cavo-tricuspid isthmus line	2 (10.5)
Ablation of autonomic Ganglia	19 (100)

**Abbreviations.** PVs: Pulmonary Veins.

Three patients (15.7%) underwent mitral isthmus line and in all cases a complete block was achieved. A cavo-tricuspid isthmus (CTI) line was carried out in two patients (10.5%) with successful bidirectional block. All surgical procedures were completed as planned without any conversion to cardiopulmonary bypass. Median operative time was 216 minutes (IQR 132–391). There were neither early deaths nor complications during the postoperative course. Median Intensive care unit (ICU) stay was 6.9 hours [IQR 4.0–14.0] and median in-hospital length of stay was 3.6 days [2.7–4.3].

No patient died during the follow up. There was a significant improvement in median EHRA (European Hear Rhythm Association) score at follow up (1[IQR 1–2], *p* < 0.001 vs. baseline). At one year, 7/19 (36.8%) patients were in sinus rhythm and off-AAD. Furthermore, 63.1% (12/19), patients were free from AF, AFL or AT >30 s but still under antiarrhythmic therapy (*p* = 0.1).

**Figure 1:**
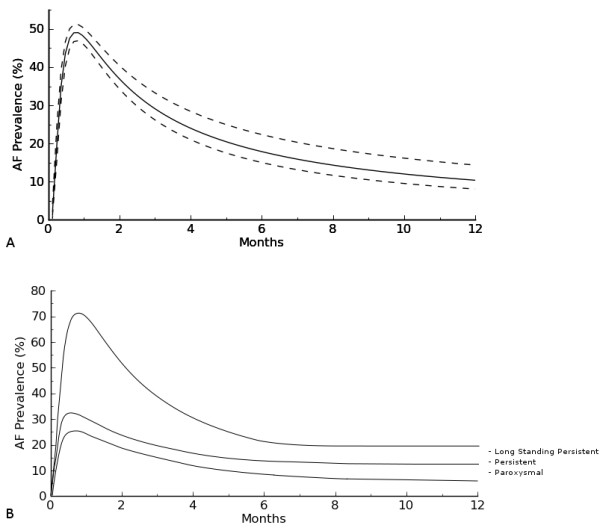
**A. Time-related prevalence of Atrial Fibrillation after hybrid procedure.B**. Time-related prevalence of Atrial Fibrillation by AF type.

**Figure 2:**
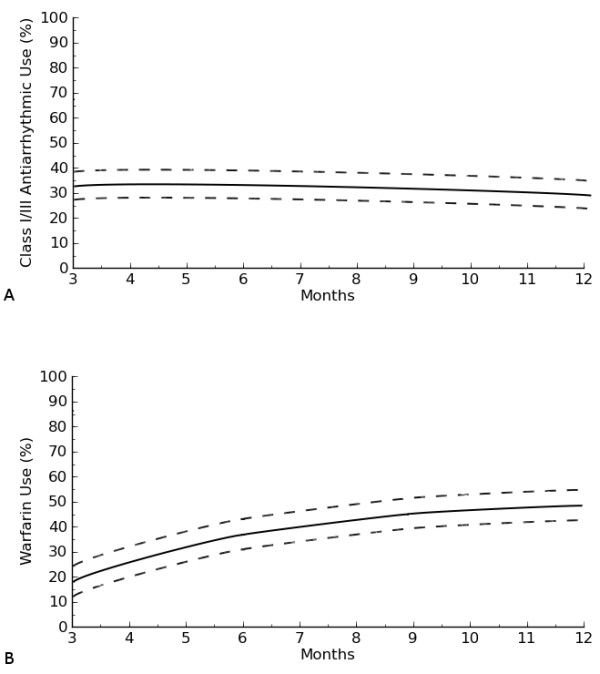
**A. Prevalence of class I/III anti-arrhythmic use after hybrid procedure.B**. Prevalence of Warfarin use hybrid procedure.

Time-related prevalence of postoperative AF was 44.4%(41.3–47.4) at two weeks, 30.4% (27.3–34.9) at three months, 14.2% (11.6–18.1) at 6 months and 13.3% (11.0–17.4) at 12 months (Figure 1A). Among patients with long-standing persistent AF, 20% (2/10) were in SR and off-AAD. One-year success rates were 50% (2/4) in persistent and 60% (3/5) in paroxysmal AF. At 12 months the estimated prevalence of antyarrhythmic drugs (Figure 2A) was 26% (22.4–33.1). AAD-prevalences by AF type were 38.1% (34.3–43.4) for long-standing persistent, 26.3% (22.2–30.7) for persistent and 14.9% (9.9–17.8) for paroxysmal AF. Finally, time-related prevalence of Warfarin (Figure 2B) at 1 year was 48.2% (44.2–52.2). This figure was 52.5% (48.8–55.2) in long-standing persistent, 35.3% (32.5–39.2) in persistent and 19.4% (16.3–23.3) in paroxysmal AF. The cumulative incidence of electrical cardioversion was 0.05/patient.

### LA function and remodeling

Table 3 shows postoperative echocardiographic data. At 3 month- follow-up biplane LAVI was reduced by 16% (*p* = 0.05). It further decreased but not significantly at 1 year (*p* = 0.09). Based on the cut-off value (≥15% reduction in LAVI) LARR occurred in 47.3% (n = 9) of patients. LA_EF_ increased not significantly at 3 month- (*p* = 0.6) and at 12-month- (*p* = 0.56) controls. Finally, LA diameters decreased significantly at 3 months (LA A-P, *p* = 0.02; LA S-I *p* = 0.03) whereas at one year there was a further reduction which did not reach statistical significance (LA A-P, *p* = 0.73; LA S-I *p* = 0.81).

**Table 3:** LA remodeling

**LAVI (mL/m**^**2**^**)**	
3 months	45 ± 10
12 months	40 ± 11
p	0.09
**LA**_**Max**_**(mL/m**^**2**^**)**	
3 months	46 ± 15
12 months	45 ± 13
p	0.7
**LA**_**Min**_**(mL/m**^**2**^**)**	
3 months	27 ± 10
12 months	25 ± 8
p	0.86
**LA**_**EF**_**(%)**	
3 months	41 ± 14
12 months	43 ± 14
p	0.56
**LA A-P (cm)**	
3 months	3.9 ± 0.4*
12 months	3.7 ± 0.4
p	0.73
**LA S-I (cm)**	
3 months	5.9 ± 0.6*
12 months	5.8 ± 0.6
p	0.81

Normal data were presented as mean ±1 Standard deviation (SD). *Significance vs. Baseline. **Abbreviations:** LAVI: (Biplane) Left atrial volume index; LAMax: Maximum left atrial volume; LAMin: minimum left atrial volume; LA_EF_ : Left atrial emptying fraction; LA A-P: Left atrial antero-posterior diameter; LA S-I: Left atrial antero-posterior diameter.

## Discussion

Minimally invasive surgical epicardial thoracoscopic approaches have gained a growing popularity but data regarding their efficacy are controversial [6-9,22-25]. The hybrid approach combines, in one step, a thoracoscopic epicardial ablation with a PCA procedure [9,26]. The surgeon, through a thoracoscopy, can isolate the pulmonary veins and the posterior wall of the LA whereas the endocardial “step” offers the possibility of evaluating the endpoints of the ablation and it gives the opportunity to add an endocardial “touch-up” in case of incomplete epicardial PVs isolation. In addition, the electrophysiologist (EP) can make lesions in regions which cannot be reached epicardially. Moreover, this technique may potentially decrease the complication rate of both surgical and catheter ablation procedures. Indeed, from the point of view of the surgeon, the risk of post-operative arrhythmias is reduced since ablation lines and lesion set can be controlled endocardially and the risk of injury is lower, since the EP can make lesions in areas which cannot be easily reached by the surgeon.

On the other hand, from the EP point of view, there is no risk of phrenic nerve and esophageal injury because these structures can be protected by the surgeon and the possibility of tamponade is low since the pericardium is open. Furthermore, by reducing the total number of endocardial ablations the risk of embolism is potentially reduced [27].

We present our one-year results with minimally invasive hybrid-right thoracoscopic approach employing a monopolar radiofrequency (RF) source for the treatment of LAF. Little information exists in the literature about the hybrid technique [10,28] and, to the best of our knowledge, no data exist on the right monolateral monopolar hybrid approach.

The monolateral right-thoracoscopic approach could theoretically reduce the rates of complications (bleeding, pulmonary complications) and significantly shorten patients' recovery time. Nonetheless, only monopolar devices can be used through the right-thoracoscopy because of the lack of maneuverability of bipolar clamps (which can hamper the orientation of the probe and constrain the variety of achievable lesion sets when employed from the right side) but many concerns have been raised regarding the ability of monopolar devices to create transmural lesion with bidirectional conduction block on the beating heart [29].

This technique demonstrated, in our experience, a high degree of safety with no major complications and no major thromboembolic events detected during the follow up period. Nonetheless, at one year only 36.8%(7/19) patients were in SR and off-AAD. Among patients with long-standing persistent AF, 20% (2/10) were in SR and off-AAD. One-year success rates were 50% (2/4) in persistent and 60% (3/5) in paroxysmal AF. Furthermore, at 12 months, the estimated prevalence of antyarrhythmic drugs was 26% (38.1% in LSP, 26.3%in persistent and 14.9% in paroxysmal AF). The incidence of patients without recurrent AF but with AAD was not significantly lower compared with patients off-AAD (*p* = 0.1) although this data must be interpreted taking into account the small number of patients in the study. Therefore, this limitation does not allow us to draw any conclusion about the role of AAD in the development of AF recurrence following minimally invasive surgery.

However, the success rate, in this series, was lower than published papers employing monopolar devices [30] and it might be explained by the high number of patients with long-standing persistent (LSP) and persistent AF which represent 73.6% of our cohort. Indeed, LSP and persistent AF may be considered to be advanced stages of the arrhythmia characterized by significant changes in the atrial tissue and muscle (substrate modification) that leads to chaotic electrical activity. Thus, these patients might need more areas in the heart ablated than in the monolateral approach and they might benefit much more from procedures which increase the likelihood that ablation lines are transmural although this aspect may be addressed by the EP who can complete any line by focused endocardial applications. Indeed, in our experience, the incidence of incomplete, non trans-mural gap lesions was high, none of the patients showed entrance and/or exit block after the epicardial ablation and,after completing PV isolation endocardially, all patients had a conduction delay > 200 ms. in the posterior LA but no patient had complete block.

With this current knowledge we have stopped using monopolar devices for the surgical treatment of AF, even for patients where a monolateral approach may have a potential advantage i.e. in case of reduced pulmonary function. In these patients we now perform a monolateral approach from the right or left side, depending on the wish to close the LAA, using bipolar devices^10^ and isolating the opposed PVs endocardially with RF energy.

Finally, at latest follow up only 47.3% of patients experienced LARR and there was no significant improvement in LAVI and LAEF. Due to the small number of patients, we could not compare echocardiographic data by AF type. However, again, the high percentage of subjects with LSP or persistent AF reflects a greater extent of substrate modifications and atrial structural remodeling and this could explain the lack of LARR. Indeed, all five patients with paroxysmal AF showed a ≥15% decrease in LAVI.

### Limitations of the study

This study has some limitations which have to be pointed out. The small patient population and the short follow-up do not allow us to draw definite conclusions. Larger series with long-term follow up are needed. Second, only a small number of patients in this series underwent right-sided and isthmus lesions and this might have influenced the results. Third, our study did not compare the hybrid right thoracoscopic approach with the hybrid bilateral thoracoscopic approach to evaluate the real impact of these different techniques on outcome of LAF patients and to evaluate whether the excision of the LAA, besides the management for prevention of thromboembolic events, might have a contribute to different rhythm outcome. Furthermore we did not compare PCA with surgical thoracoscopic approaches. However, all these aspects will be the subjects of ongoing studies.

Fourth, while data regarding postoperative AF prevalence were obtained from a large number of observations and this allowed us to compare prevalence by AF type, we could not evaluate LAVI, LAEF and LA diameters in any subgroup of patients.

Finally, atrial function was studied employing only the emptying fraction and neither pulsed-wave Doppler nor Tissue Doppler Imaging (TDI) nor LA strain were employed, which would have given more detailed information about LA function after AF surgical ablation. All these aspects deserve further investigations

## Conclusions

One year results combining the percutaneous endocardial with the right thoracoscopic epicardial technique were, in our experience, not satisfactory, particularly in patients with LSP and persistent AF. This approach might still be considered a possible alternative to PCA in selected patients with paroxysmal lone atrial fibrillation and impaired pulmonary function referred to surgery when the LAA excision/ligation is not deemed necessary. Our findings need to be confirmed by larger studies.

## Abbreviations

LAF, Lone Atria Fibrillation; PCA, Percutaneous Catheter Ablation; RF, Radiofrequency; LSP, Long-standing persistent; EHRA score, European Heart Rhythm Society score; SCV, Superior caval vein; ICV, Inferior Caval Vein; LAA, Left Atrial Appendage; GP, Ganglionated plexi; PVs, Pulmonary Veins; CTI, Cavo-tricusoid isthmus (lesions); CS, Coronary sinus; SR, Sinus Rhythm; EP, Electrophysuologist; LARR, Left Atrial Reverse Remodeling; LAVI, (Biplane) Left atrial volume index; LAMax, Maximum left atrial volume; LAMin, Minimum left atrial volume; LAEF, Left atrial emptying fraction; LA A-P, Left atrial antero-posterior diameter; LA S-I, Left atrial antero-posterior diameter.; IQR, Interquartile Range; TEE, Transesophageal Echocardiography; TTE, Transthoracic echocardiography.

## Competing interests

Dr La Meir has been consultant/advisor for Atricure and Estech. Other co-authors do not have any conflict of Interest.

## Authors’ contribution

MLM Research design, drafting, approval Equal Contributors. SG Research design, drafting, approval Equal Contributors. RL Research design, drafting, approval. FL Article Collection, Research design, drafting, approval. LP Critical Revision, drafting, approval. OP Article Collection, Research design, drafting, approval. FW Critical Revision, drafting, approval. GFG Drafting, Final revision and approval of the manuscript. JM Final revision and final approval of the manuscript. All authors read and approved the final manuscript.
